# Hypocalcemia in Malignancy - Unexpected but Common

**DOI:** 10.7759/cureus.442

**Published:** 2015-12-31

**Authors:** Bobby Diniotis, Evan Sternberg, Shrestha Shakuntala, Maguy Chiha, Pam Khosla

**Affiliations:** 1 Internal Medicine, Mount Sinai Chicago; 2 MS4, Mount Sinai Chicago; 3 Oncology Department, Mount Sinai Chicago; 4 Endocrinology Department, Mount Sinai Chicago; 5 Hematology and Oncology Department, Mount Sinai Chicago

**Keywords:** hypocalcemia, non-hogkins lymphoma, vitamin d deficiency, r-chop, folfox, symptoms of hypocalcemia, hypocalcemia in malignancy

## Abstract

One of the most common metabolic abnormalities found in patients with malignancy is hypercalcemia. Hypocalcemia is a rare occurrence and is often found to be associated with renal failure and patients taking bisphosphonate therapy for bone metastasis in this patient population. Here, we present two different case reports with hypocalcemia. A 66-year-old female with a recent diagnosis of tonsillar diffuse B-cell lymphoma admitted with complaints of generalized weakness after one cycle of R-CHOP, found to have neutropenia, a low calcium level, high PTH, and low 25-hydroxy Vitamin D levels. She was given calcium gluconate and supplemental 25-hydroxy Vitamin D. On day 2, the patient's symptoms and counts improved. The second patient was a 64-year-old male with recurrent metastatic laryngeal carcinoma, along with a second locally advanced primary rectal adenocarcinoma, presented with severe hypocalcemia and a low PTH level. The patient was on adjuvant chemotherapy and exhibited Chvostek’s sign, along with perioral numbness, tingling, and twitching sensations, which eventually led to dysphagia. He was treated with calcium gluconate, calcitriol, and calcium carbonate. Signs and symptoms, along with lab values, improved on day 4. These cases suggest that calcium kinetics and 25-hydroxy Vitamin D levels need to be monitored in these patient populations in a routine manner.

## Introduction

Hypocalcemia is a condition which has many causes, such as low calcium intake, vitamin D deficiency, abnormal magnesium or phosphate levels, and inadequate parathyroid hormone (PTH) levels [1]. Hypocalcemia is also prevalent in certain cancers via mechanisms pertaining to osteoblastic-stimulating factors or toxic effects of certain chemotherapeutic agents. The most well-known chemotherapy drug known to cause hypocalcemia is cisplatin, which results in low calcium secondary to hypomagnesemia [2]. One of the most common causes of low calcium level is actually known as pseudo-hypocalcemia. This is a condition is where other factors, such as low albumin concentration, result in a falsely low level of calcium. In this condition, the calcium level may actually be normal once it has been corrected for the abnormality in albumin. Another common cause of low calcium is from a hormonal imbalance. The major hormones that are responsible for calcium regulation are PTH and Vitamin D via the effects on bones, kidneys, and gastrointestinal (GI) tract [1]. Vitamin D deficiency plays a role in calcium concentrations resulting in secondary hyperparathyroidism. The most common cause of Vitamin D is low intake, as foods are not commonly fortified in 25-hydroxy Vitamin D. Low Vitamin D levels cause a secondary elevation in PTH levels, aiming to normalize the calcium level and prevent hypocalcemia. Hypomagnesemia can cause PTH resistance and, thus, hypocalcemia despite elevated PTH levels. Additionally, surgical, autoimmune, or post-neck radiation destruction of the parathyroid glands can result in low PTH and subsequent low calcium levels [3].

## Case presentation

Informed patient consent was obtained from both patients described in this paper.

### Case 1

A 66-year-old woman with a past medical history of lupus, Sjogren's disease, hepatitis C virus, and diffuse large B-cell lymphoma involving the right tonsil presented to the oncology clinic with increasing weakness, headache, and severe pains across her whole body one week after she received her first cycle of rituximab, cyclophosphamide, doxorubicin, vincristine, and prednisone (R-CHOP). Physical exam revealed bilateral thigh pain, right shoulder pain, and chronic right foot numbness. Bilateral lower extremity duplex was negative, and the chest x-ray showed no acute findings. The patient was found to have an absolute neutrophil count of 0.1, a potassium level of 2.8 meq/L, a calcium level of 6.1 mg/dL (corrected calcium 7.30 mg/dL), an albumin level of 2.5 g/dL, severe 25-hydroxy Vitamin D deficiency (18 ng/mL), an elevated PTH (102.9 pg/mL), a phosphate level of 2.7 mg/dL, a magnesium level of 2.1 mg/dL, a creatinine level of 0.69 mg/dL, and a GFR greater than 60. The patient was started on prophylactic antibiotics (vancomycin and cefepime), given 2 grams of calcium gluconate, potassium (total of 80 meq), and Vitamin D supplementation. On day 2, her symptoms were resolved, she had remained afebrile, calcium levels improved to 8.6 mg/dL, and the ANC increased to 0.3. On discharge, the patient was given Vitamin D, calcium supplementation, and given a follow-up appointment in order to continue to monitor her electrolytes.

### Case 2

In the next case, a 64-year-old male with a past medical history of laryngeal cancer status-post chemo/radiation in 2011 and laryngectomy as well as rectal adenocarcinoma in 2014 status-post neoadjuvant chemotherapy was admitted for neutropenic fever with a Tmax of 103.0 F, a white count of 0.8, and an ANC of 0.2. The patient was on adjuvant chemotherapy with Erbitux and (FOLFOX) 5-flourouracil, leucovorin, and oxaliplatin. The patient had a significant past medical history as stated above, along with hypoparathyroidism thought to be secondary to either radiation therapy in 2011 and/or a surgical complication from the laryngectomy. Upon admission, he was immediately started on vancomycin and cefepime. Blood cultures revealed gram-positive cocci. The patient's only complaint on admission was vomiting and abdominal pain for two days. His physical examination was pertinent for Chvostek's sign, perioral numbness, and twitching. Laboratory results showed a calcium level of 5.6 mg/dL, (corrected calcium at 6.56 mg/dL), an albumin level of 2.8 g/dL, a phosphorus level of 2.2 mg/dL, a magnesium level of 1.6 mg/dL, and a 25-hydroxy Vitamin D level of 29 ng/mL. An EKG showed QTc prolongation (490 ms). The patient was given IV calcium gluconate, calcium carbonate 1,250 mg three times a day orally, and calcitriol, 0.5 mcg daily PO, with an improvement in his signs and symptoms by the fourth day.

## Discussion

It is important to consider Vitamin D deficiency, hypomagnesemia, and chronic kidney disease in patients with hypocalcemia [4]. Hypocalcemia is a condition which is associated with a wide spectrum of clinical manifestations. Furthermore, the hallmark of the condition is tetany, which can present as perioral numbness, paresthesia, muscle cramps, carpopedal spasms, laryngospasms, or seizures. Electrocardiogram abnormalities and psychological manifestations can be present. The severity of symptoms is usually related to the acuteness of the development of hypocalcemia. Tetany is a very unusual symptom to observe among patients with chronic renal failure as their hypocalcemia is less severe and more indolent. Additionally, it is thought to be partially due to the protective effects of metabolic acidosis seen in chronic kidney disease. The most important pathophysiologic effect of hypocalcemia is from hyperexcitability of peripheral neurons, which occurs at all levels of nervous system, including motor end plates, spinal reflexes, and the CNS [5]. The two most well-known findings in a patient with hypocalcemia are Trousseau's and Chvostek's signs. Trousseau's sign is carpopedal spasms when a blood pressure cuff is inflated above the patient's systolic BP. Chvostek's sign is facial muscle contraction when the facial nerve is tapped just anterior to the ear. Often, these signs and symptoms are not observed or sought, and the sole presenting symptom is a seizure. From a psychiatric perspective, a low calcium level can cause emotional instability, anxiety, and depression before any external manifestation arises. Vitamin D deficiency can present with muscle weakness and hypotonia. Due to the wide variety of signs and symptoms, it is necessary to check calcium, magnesium, phosphate, PTH, and 25-hydroxy Vitamin D in any patient with suspected hypocalcemia. 

## Conclusions

In conclusion, hypocalcemia is a complication as well as a comorbidity of many cancer treatments. It can be a preexisting comorbidity if the patients have unrecognized and untreated 25-hydroxy Vitamin D deficiency as well as malnutrition and calcium deficiency. It can be a complication of treatment, such as neck dissection and neck radiation, causing hypoparathyroidism. Certain chemotherapeutic agents are also associated with electrolyte imbalances that can cause PTH resistance and, thus, hypocalcemia. Lastly, treatment of bone metastasis with anti-resorptive agents can be associated with hypocalcemia. Hypocalcemia can be as serious as hypercalcemia, especially if acute in onset, but when insidious, it can go unrecognized until severe. Symptoms can be of a wide variety, can range from mild to severe, and include psychological disturbances, such as, but not limited to, seizures and arrhythmias. Prompt recognition is essential and prevention is also crucial; all cancer patients should have adequate 25-hydroxy Vitamin D levels and calcium intake.
